# Modified mandible traction with wires to treat neonatal Pierre Robin sequence: A case report

**DOI:** 10.3389/fsurg.2022.899195

**Published:** 2022-09-22

**Authors:** Hailiang Zuo, Jing Gao, Yu Mu, Fang Zhang, Yang Liu

**Affiliations:** ^1^ Department of Plastic Surgery, Tianjin Children's Hospital/Tianjin University Children's Hospital, Tianjin, China; ^2^ Department of Neonatology, Tianjin Children's Hospital/Tianjin University Children's Hospital, Tianjin, China; ^3^ Graduate College, Tianjin Medical University, Tianjin, China

**Keywords:** Pierre Robin sequence, mandible traction, wire, neonate, micrognathia, upper airway obstruction

## Abstract

**Background:**

Pierre Robin sequence (PRS) is a congenital craniofacial deformity characterized by micrognathia, glossoptosis and airway obstruction. Some affected neonates are born with severe life-threatening upper airway obstruction that requires surgery. If without timely treatment, it is possible to cause not only organ damage and developmental abnormalities but also early newborn mortality.

**Case presentation:**

In this report, a 51-hours-old neonate was diagnosed with PRS, who had severe upper airway obstruction and required surgery. We performed the modified mandible traction with wires at four days old and achieved a satisfactory result in improving airway obstruction. No other complications were observed except for mild local infection. No overlap of other more complex syndromes was found, such as ocular abnormalities, hearing loss, other skeletal abnormalities, cardiac abnormalities or other atypical abnormalities. At the present follow-up until 2 years old, there were no significant differences in the maxillofacial appearance, teeth growth, breathing, feeding, growth and development between the patient and normal children.

**Conclusion:**

The modified mandible traction with wires can safely and effectively resolve micrognathia, the key to treating PRS, which is minimally invasive, simple and provides immediate relief of airway obstruction with no long term complications compared with other surgical methods. This report aims to provide more evidence of the successful treatment of neonatal PRS micrognathia by modified mandible traction with wires.

## Introduction

Pierre Robin sequence (PRS) is a congenital craniofacial deformity characterized by micrognathia, glossoptosis and airway obstruction (1). In addition, PRS also had a wide cleft palate, which is not requisite for the diagnosis but is found in up to 85% ∼ 90% of PRS patients (2, 3). About 50% PRS patients overlap with other more complex syndromes, such as Stickler syndrome, Velo-cardio-facial syndrome, Treacher-Collins syndrome and so on (2). The combination of the small and receding mandible, tongue falling back, and upper airway obstruction make a cascade reaction of respiratory distress and feeding difficulties at the birth of PRS neonates. Therefore, lengthening mandible to solve mandibular deficiency and restore normal morphology of mandible is the key to treatment (4). Surgical therapies include tongue lip adhesion (TLA), mandible traction, and tracheostomy (5, 6). Although each surgical method has been applied in the neonatal period, they have different limitations and risks (4, 7–11). Existing researches lack a consensus on optimal surgery for neonatal PRS. In this report, a 51-hour-old PRS neonate was admitted because of respiratory distress, and physical examination revealed a wide cleft palate, micrognathia, retrognathia, glossoptosis, and cyanosis. Arterial oxygen saturation was 80%. Computed Tomography (CT) examination showed distance between the base of tongue and posterior pharynx wall was narrowed significantly about 1.4 mm. The modified mandible traction with wires successfully and safely treated the neonate. We recorded treatment process and follow-up in detail, expecting to provide more references for the surgical treatment of neonatal PRS.

## Case report

**Figure 1:**
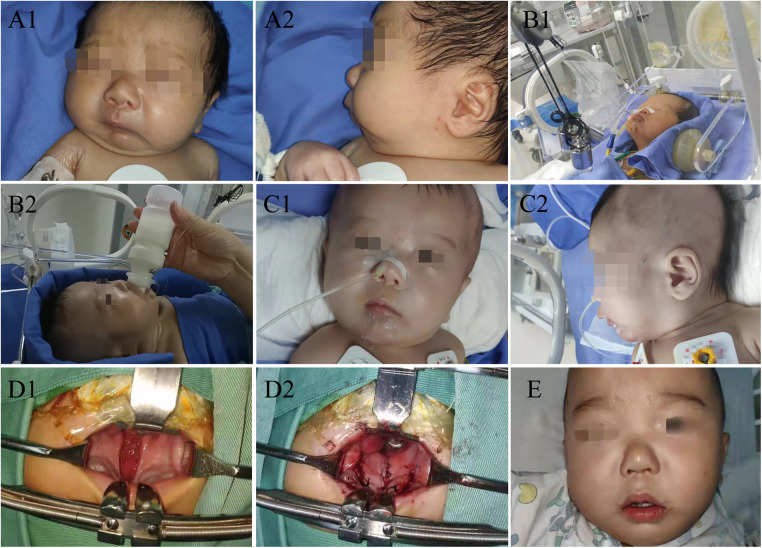
(**A1**) Front view on admission; (**A2**) side view on admission; (**B1**) traction; (**B2**) oral feeding in traction; (**C1**) front view after removing the wire; (**C2**) side view after removing the wire; (**D1**) cleft palate repair; (**D2**) cleft palate repair; (**E**) after cleft palate repair, visible the central incisors and lateral incisors.

**Figure 2:**
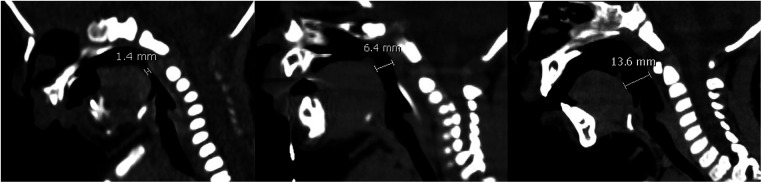
From left to right are CT images on preoperative day 1, postoperative day 28, postoperative 1 year 2 months. CT images showed the distance between the base of tongue and posterior pharynx wall was 1.4 mm, 6.4 mm, 13.6 mm.

A 51-hour-old male neonate was admitted to the Neonatal Intensive Care Unit because of respiratory distress and feeding difficulties on June 30, 2020. The neonate was full-term, delivered by cesarean section, and with low birth weight was 2,450 g. Physical examination revealed a wide cleft palate, micrognathia, retrognathia, glossoptosis and cyanosis (Figure 1A). Respiratory rate was 64 breaths/min, and arterial oxygen saturation was 80%. CT examination showed distance between the base of tongue and posterior pharynx wall was narrowed significantly (about 1.4 mm) with narrowing of the upper airway at the corresponding level (the second cervical vertebra to the fourth cervical vertebra), shortened and retracted mandible (Figure 2). Both clinical manifestations and CT images of this neonate showed prominent features and met the diagnostic criteria of PRS. At the meantime, no overlap of other more complex syndromes was found, such as ocular abnormalities, hearing loss, other skeletal abnormalities, cardiac abnormalities or other atypical abnormalities.

After the definite diagnosis, we performed modified mandible traction with wires for the neonate on July 02, 2020, because prone position did not completely relieve the symptoms of hypoxia due to airway obstruction. Four puncture points were marked approximately 2 mm near the upper and lower margins of the mandible, symmetrically on both sides, and about 8 mm from the midline. The neonate was placed in supine position and disinfected with iodophor according to routine. After spreading out drapes and disinfecting again, 0.25% lidocaine was injected along surgical area for local anesthesia. After anesthesia, a needle with wire was inserted from outside of mouth close to the lower margin of mandible to avoid damaging the sublingual gland, submandibular gland and other tissues. Then the needle was pulled out from inner side and through the homolateral upper marked point from inside out. The same procedure was performed on the other sides. Use epinephrine saline gauze to press wound to stop bleeding completely. Intraoperative blood loss was about 5 ml, and the operation took 25 min with a satisfactory result in improving airway obstruction.

After surgery, the neonate's vital signs were stable; arterial oxygen saturation increased to 99%. Mandible was hauled continuously with external wire traction equipment, and traction weight was gradually reduced from 150 g until the traction wire was removed 35 days later (Figures 1B,C). In this period, only slight superficial skin infection at puncture sites was found, which could be controlled by topical application of antibiotic ointment and infusion of antibiotics. CT examination was repeated on postoperative day 6, 11, 16, 28 showed the distance between the base of tongue and posterior pharynx wall was 6.6 mm, 6.7 mm, 6.7 mm and 6.4 mm (Figure 2). The child was hospitalized for 38 days and discharged with a weight of 2.8 kg. The child performed cleft palate repair at 1 year and 2 months. CT examination showed the distance between the base of tongue and posterior pharynx wall was 13.6 mm (Figure 2). At the present follow-up until 2 years old, there were no significant differences in the maxillofacial appearance, teeth growth, breathing, feeding, growth and development between the patient and normal children.

## Discussion

Pierre Robin sequence (PRS) is a congenital disease first proposed by French stomatologist Pierre Robin in 1923, which is characterized by micrognathia, glossoptosis and airway obstruction (1). PRS may cause not only organ damage and developmental abnormalities but also early infant death. Although the pathogenesis remains unclear, it is definite that cascade reaction of micrognathia, glossoptosis and upper airway obstruction make respiratory distress and subsequent feeding difficulties. Upper airway obstruction greatly disturbs the order of sucking, breathing, and swallowing. Besides, keeping airway open while feeding increases calorie consumption significantly (7). Therefore, lengthening mandible to restore normal shape can relieve airway obstruction and overcome feeding difficulties, which is the key to treating PRS (4).

The treatment of PRS is divided into non-surgical and surgical. Non-surgical treatment includes positioning therapy, nasopharyngeal airway, tracheal intubation, etc. Prone or lateral position is the most simple, convenient, easy to implement, and can resolve mild airway obstruction. But it is inefficient and uncertain and can not solve severe PRS. Nasopharyngeal airway impedes feeding and is prone to dislocation, which may lead to nausea, vomiting even suffocation. Tracheal intubation is definitely effective in improving ventilation, but it is generally used as a short-term and emergency measure because of its invasiveness.

Surgery can be selected when non-surgical treatment fails, including tracheostomy, tongue lip adhesion (TIA), and mandible traction. Although each surgical method has been applied in the neonatal period, due to different limitations and risks, there is still a lack of consensus on optimal surgery for PRS (12).

Tracheostomy causes irreversible damage to trachea and is associated with significant morbidity and mortality. In addition to a potential risk of accidental decannulation and mucous plugging, there may be narrowing of trachea related to peristomal scarring and tracheal erosion (8, 9). It also has a longer hospital stay, higher charges and incidence of gastrostomy feeding (8). Therefore, tracheotomy is rarely used in current clinics. TIA acts to increase cross-sectional area of oropharyngeal airway by anchoring backward displaced tongue base to the lower lip or hypoplastic mandible. It relies on the controversial “catch-up growth” theory of mandible to eventually ease airway obstruction (4, 10). Some researchers found mandible structures did not reach normal values compared with control groups (4, 10). Besides, a researcher suggests TIA should be abandoned because it has the same group of beneficiaries as nasopharyngeal airway (7).

More and more surgeons now choose mandible traction as preferred surgery because of its advantages (8, 13–17). One is mandibular distraction osteogenesis (MDO), published by McCarthy et al. in 1992 (5); the other is mandible traction with wires, described by Baciliero et al. in 2011 (6). Ilizarov applied bone lengthening to endochondral bone of the extremities in 1951, then other researchers applied the technique to the mandibles of animals in 1973, and eventually McCarthy successfully extended it to the human mandible by MDO (18). At present, MDO is a more mainstream method of surgical traction, which lengthens mandible by using a traction device after truncating mandible. But it has great surgical damage, a high risk of general anesthesia and complications (11). Injury to tooth buds and facial nerves, especially permanent dentition, is difficult to avoid and predict (19). Mandible traction with wires can maintain the integrity of mandible and has a good safety profile compared to MDO (20). This patient's surgical and follow-up results also support this view. Meanwhile, simpler procedures, earlier oral feeding, and fewer complications may help to reduce economic burden on families and society.

This report chose modified mandible traction with wires to treat neonatal PRS. Compared with the procedure of Baciliero et al. and Dong et al. (6, 20), it has some obvious differences in puncture points, traction direction, position, and feeding patterns. Four puncture points passed through mandible can reduce injury of mandibular margins and tooth buds caused by tied wires. The different traction direction due to higher puncture points avoided mandible closing to chest wall and was conducive to oral feeding and swallowing. Keeping the supine position also facilitated oral feeding without stopping traction. Nasogastric tube and oral feeding were started simultaneously on postoperative day 2 to ensure adequate calorie intake is shown in Supplementary Video 1. Dong et al. proposed that constant traction in a fixed lateral recumbent position may lead to plagiocephaly and sensory deprivation (20). However, this patient without sensory deprivation during follow-up. Subsequent CT examination also showed no plagiocephaly or skeletal malocclusion. It is reasonable to speculate that supine position and oral feeding may somewhat avoid these potential limitations to some extent. In addition, respiratory-related indexes monitored in the supine position were more actual without interference from lateral position. All modifications made nursing easier without changing position, regular oral care and suctioning oral secretions. Initial traction weight was increased to 150 g, partly because of tongue drooping in the supine position, and partly to shorten the time and reduce the risk of fractures due to long-term traction.

The neonate had no other complications except slight superficial skin infection at puncture sites that could be controlled by topical application of antibiotic ointment and infusion of antibiotics. At present stage of follow-up, there were no significant differences in the maxillofacial appearance, teeth growth, breathing, feeding, growth and development between the patient and normal children (Figure 1E). We consider early and effective modified mandible traction with wires is the key to restoring normal growth of mandible. In particular, this intervention did not affect growth rate or quality of teeth and has many advantages.

## Conclusion

Compared with other methods, modified mandibular wire traction has changed in terms of puncture points, traction direction, position and feeding patterns. It not only addresses micrognathia and retrognathia directly but also has advantages of no impact on teeth growth, a simple procedure, little trauma, low risk of local anesthesia, fewer complications, simple nursing, convenient feeding and so on. It may be a safer and more effective surgery to treat PRS neonates early. The report records treatment process in detail, expecting to provide more references for surgical treatment of PRS in the neonatal period.

## Data Availability

The original contributions presented in the study are included in the article/Supplementary Material, further inquiries can be directed to the corresponding author/s.

## Supplementary material

Supplementary Video 1 The video of oral feeding while in traction at 28 days postoperatively.
